# *In Vivo* MRI of Functionalized Iron Oxide Nanoparticles for Brain Inflammation

**DOI:** 10.1155/2018/3476476

**Published:** 2018-06-25

**Authors:** Tang Tang, Anthony Valenzuela, Fanny Petit, Sarah Chow, Kevin Leung, Fredric Gorin, Angelique Y. Louie, Marc Dhenain

**Affiliations:** ^1^Chemistry Graduate Group, University of California, Davis, CA 95616, USA; ^2^Department of Neurology, School of Medicine, University of California Davis, 4860 Y Street, Sacramento, CA 95817, USA; ^3^Centre National de la Recherche Scientifique (CNRS), Neurodegenerative Diseases Laboratory, Université Paris-Sud, Université Paris-Saclay, UMR 9199, 92260 Fontenay-aux-Roses, France; ^4^Commissariat à l'Energie Atomique et aux Energies Alternatives (CEA), Direction de la Recherche Fondamentale (DRF), Molecular Imaging Research Center (MIRCen), 92260 Fontenay-aux-Roses, France; ^5^Department of Biomedical Engineering, University of California, Davis, CA 95616, USA

## Abstract

Microglia are intrinsic components of the brain immune system and are activated in many central nervous system disorders. The ability to noninvasively image these cells would provide valuable information for both research and clinical applications. Today, most imaging probes for activated microglia are mainly designed for positron emission tomography (PET) and target translocator proteins that also reside on other cerebral cells. The PET images obtained are not specific for microglia-driven inflammation. Here, we describe a potential PET/MRI multimodal imaging probe that selectively targets the scavenger receptor class A (SR-A) expressed on activated microglia. These sulfated dextran-coated iron oxide (SDIO) nanoparticles are avidly taken up by microglia and appear to be nontoxic when administered intravenously in a mouse model. Intravenous administration of this SDIO demonstrated visualization by T_2_^*∗*^-weighted MRI of microglia activated by intracerebral administration of tumor necrosis factor alpha (TNF-*α*). The contrast was significantly enhanced by SDIO, whereas there was little to no contrast change in animals treated with nontargeted nanoparticles or untreated controls. Thus, SR-A targeting represents a promising strategy to image activated microglia in the brain.

## 1. Introduction

Inflammation occurs in various central nervous system diseases, such as neurodegenerative diseases, epileptic disorders, as well as autoimmune diseases. Microglia, the resident macrophages in the brain, are recognized as the prime components of an intrinsic brain immune system and are the key cellular mediators of neuroinflammatory processes [1]. The activation of microglia can be triggered by an assortment of inflammatory stimuli to the central nervous system (CNS) that includes stroke, seizures, Alzheimer's disease (AD), traumatic brain injury, and CNS storage diseases [2–6]. Activated microglia are capable of synthesizing a broad range of proinflammatory and anti-inflammatory cytokines and other molecular mediators [7] that can lead to changes in the permeability of the blood-brain barrier followed by infiltration of peripheral immune cells into the CNS, increased secretion of inflammatory cytokines, exacerbated tissue damage, and neuron loss [8]. Thus, noninvasive methods for identifying activated microglia *in vivo* could lead to earlier detection of neuroinflammation and provide a means for monitoring disease progression and therapeutic intervention.

Earlier efforts have reported the development of *in vivo*, neuroinflammatory PET diagnostic agents targeting activated microglia through the peripheral benzodiazepine receptor (PBR), now known as the 18 kDa translocator protein (TSPO). ^11^C-PK11195 is one of the most widely used ligands for PET imaging of microglia in patients with neurological disorders, but this first-generation TSPO radiotracer demonstrates poor signal-to-noise ratio and poor selectivity [9, 10]. Newer tracers, such as ^11^C-PBR28, with improved TSPO binding or selectivity have been introduced and initiated in clinical studies [11–13]. Unfortunately, TSPO genetic polymorphism can adversely affect the binding of ^11^C-PBR28 leading to signal variability [14, 15]. The density of TSPO in the brain is very low, even in a neurodegenerative setting [16], and the presence of TSPO on other brain cell types reduces the specificity of TSPO [17] and its PET signal coregistered with both activated microglial cells and reactive astrocytes following LPS induced inflammation [10]. A more selective biomarker for activated microglia that could be visualized by PET or high-resolution MRI would be of therapeutic value.

Instead of using TSPO as a target, we labeled activated microglia through the scavenger receptors class A (SR-A). SR-A type scavenger receptors have been shown to be upregulated on activated microglia in pathological states and not present on quiescent adult microglia [18]. SR-A initiates inflammatory responses by recognizing pathogen- or damage-associated molecular patterns (DAMPs) [19]. SR-A expression is associated with the activation/polarization of the microglia. Because of their role in inflammation, SR-A receptors are not downregulated with ligand concentration. They also mediate very efficient and rapid internalization of bound ligand [20] and repeatedly recycle through the endosomal compartment, illustrating desirable traits as targets for labeling [21–24].

Previously, our group has developed iron oxide-based PET/MRI probes that are targeted to activated macrophages through the SR-A type scavenger receptors to image vulnerable atherosclerotic plaques [25–27]. Given the presence of this same receptor on activated microglia, in this work we investigate an improved imaging probe and demonstrate the ability to specifically label and image activated microglia in a mouse model of brain inflammation [28]. The probe is sulfated dextran-coated iron oxide (SDIO) nanoparticles conjugated with the macrocyclic chelator 1,4,7,10-tetraazacyclododecane-1,4,7-triacetic acid (DO3A); the surface sulfation allows for specific targeting to SR-A. Sulfated dextran (a.k.a. dextran sulfate) is widely known as one of the ligands for SR-A with a *K*_d_ of 3 nM [29]. In our previous study, we have confirmed that the sulfation of dextran coating significantly improved the cellular binding and uptake of these nanoparticles by macrophages and the uptake efficiency enhances with higher level of surface sulfation [30]. The sulfated dextran-coated iron oxide nanoparticles have a similar binding affinity to the SR-A, compared with dextran sulfate itself. Thus, we hypothesize that these particles will also serve as promising imaging probes to visualize activated microglia *in vivo*. From the series of nanoparticles that we reported in previous work, SDIO-DO3A-10 had the highest surface sulfation level and best uptake efficiency [30]. SDIO-DO3A-10 and the nonsulfated analog DIO-DO3A are used in this study and referred to as SDIO and DIO, respectively. Here, we demonstrate the specific targeting of SDIO to activated microglia *in vitro* and their efficient MRI contrast enhancement ability in a mouse model of brain inflammation as a first step in validation for microglia imaging.

## 2. Materials and Methods

### 2.1. General Materials

Materials were purchased from commercial suppliers and used directly, unless specifically noted. Human TNF-*α* was purchased from PeproTech Inc. (Rocky Hill, NJ). Sulfated dextran-coated iron oxide was synthesized as described in our previous paper [30]. Lipopolysaccharides were purchased from Sigma-Aldrich (St. Louis, MO). Fetal bovine serum (FBS), L-glutamine, and Dulbecco's phosphate-buffered saline (DPBS) (1x) were from GIBCO. Lipoprotein-deficient bovine serum (LPDS) was obtained from Biomedical Technologies, Inc. (Stoughton, MA). Murine BV2 microglia were obtained from American Type Culture Collection (ATCC). Saline and isoflurane were acquired from APP Pharmaceuticals, LLC (Schaumburg, IL), and Piramal Healthcare Limited, respectively. Buprenex Injectable was received from Reckitt Benckiser Healthcare Ltd. Beuthanasia-D Special was purchased from Schering-Plough Animal Health Corp.

### 2.2. Syntheses of DIO and SDIO

Nanoparticles were synthesized and characterized as previously described in detail [30]. Briefly, dextran-coated iron oxide (DIO) nanoparticles were synthesized by coprecipitation of iron salts and reduced dextran with the addition of ammonium hydroxide. Purified DIO was conjugated with the DO3A chelator, which has high stability for copper-64 ions. This generated multimodal function for either PET or MRI applications. Conjugation of the chelator is a two-step reaction directly coupling to the hydroxyl groups in the dextran coating, and we sulfated the dextran coating after conjugation of the chelator to maximize the sulfate level on the surface. The highest sulfation level was used to synthesize SDIO nanoparticles. Extensive physical characterizations such as dynamic light scattering for hydrodynamic size distribution, transmission electron microscopy (TEM) for iron oxide core size, atomic absorption spectroscopy (AAS) for iron content percentage, combustion infrared for sulfur content percentage, and relaxivity measurements were performed on both nonsulfated precursor DIO and SDIO nanoparticles.

### 2.3. Biocompatibility

Biocompatibility of SDIO on murine BV2 microglia was evaluated using C_12_-resazurin viability assays. BV2 cells were maintained in Dulbecco's Modified Eagle Medium (DMEM) containing 10% fetal bovine serum (FBS) and penicillin-streptomycin (100 U/mL) at 37°C in a humidified 5% CO_2_ atmosphere. To perform the viability experiments, BV2 cells were plated in 96-well plates at a concentration of 10^4^ cells per well and incubated in a 5% CO_2_ atmosphere at 37°C overnight. The medium was then replaced with fresh media containing varying concentrations of SDIO (at 0.04, 0.2, 1, 4, 10 mM [Fe]) and incubated for 4 or 24 h. The medium was then removed, and the cells were washed with Dulbecco's phosphate-buffered saline solution (DPBS) three times. Media containing C_12_-resazurin (5 *μ*M) was then added to the wells, and after a 15 min incubation, fluorescence was measured on a Safire2 monochromator microplate reader (Tecan Austria, Groedig, Austria) with excitation at 563 nm and emission at 587 nm.

### 2.4. Cellular Uptake Studies

To study the specific cellular uptake of SDIO nanoparticles by microglia, BV2 cells (between passages 20 and 40) were grown/maintained in Dulbecco's Eagle's minimum essential medium (DMEM) containing 10% fetal bovine serum (FBS) and penicillin-streptomycin (100 U/mL) in 5% CO_2_ and then plated in 6-well plates at a concentration of 1 × 10^6^ cell/well (2 mL). After reaching ∼80% confluency, plated cells were rinsed twice with Dulbecco's phosphate-buffered saline solution (DPBS) and activated through exposure to lipopolysaccharide (LPS) in serum-free media at a final concentration of 200 ng/well for 6 h. Activated BV2 cells were incubated with DIO or SDIO solutions at different concentrations (25, 50, and 100 *μ*M iron) for 1 h. Plated cells were then rinsed three times with DPBS. Deionized water (1 mL) was then added, and the freeze/thaw (30 min/20 min) method was repeated twice to lyse cells. Cell lysates were lyophilized, and DI water (0.3 mL) was added to the residue to prepare the solution for T_2_ measurement by the Bruker Minispec mq60. T_2_ values were measured using a Carr–Purcell–Meiboom–Gill sequence, with *τ* = 1 ms and 200 data points.

### 2.5. *In Vivo* Study

**Figure 1:**
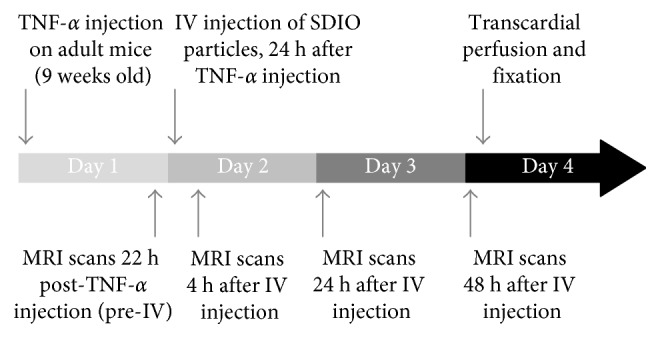
Overview of the *in vivo* study timeline.

All animal experiments were performed under a protocol approved by the UC Davis Institutional Animal Care and Use Committee (UCD Institutional # 18025). BALB/c mice (18–22 g, 9 weeks old) were obtained from Charles River Laboratories, Wilmington, MA. SDIO and DIO were evaluated after intravenous (IV) administration in mouse models of cerebral inflammation and in control animals. Brain inflammation was induced by unilateral intracerebral injection of human TNF-*α* according to a protocol previously reported in the literature [28]. Four experimental groups were evaluated: (1) TNF-*α* only (*n*=4), (2) TNF-*α* + IV-SDIO (*n*=4), (3) TNF-*α* + IV-DIO (*n*=4), and (4) IV-SDIO only (*n*=4). Mice in group 1 received intracerebral inoculation of TNF-*α* to assess whether the chemically induced inflammation in the brain produced endogenous contrast changes. The ability of intravenous SDIO or DIO to enhance contrast on MR images of inflamed brains was tested in the groups 2 and 3. Group 4 was designed to evaluate the impact of SDIO on the normal brain. The timeline of this study is shown in Figure 1.

The unilateral intracerebral injection protocol consisted of isoflurane anesthesia (1–5% inhalation route, typically at 3% induction, 1.5–2% maintenance) of animals and placement into a stereotactic device maintained at 37 ± 0.2°C on a feedback-controlled heating pad. The scalp was shaved and prepared using 3 cycles of betadine and isopropyl alcohol prior to any incisions. A small burr hole was drilled on the left side into the skull of the mice, and TNF-*α* (1 *μ*g in 1 *μ*L) was injected (at coordinate 0.5 mm anterior, 2.0 mm lateral, and −3 mm ventral to the bregma) through a 26-gauge needle within a period of 3 minutes [28]. The opening was sutured using an Ethilon nylon suture with 18″ and P-3 reverse cutting. Buprenex (0.1 mg/kg) was administered subcutaneously at the end of the procedure and then 2 times daily for 48 h. The animals were monitored regularly for pain or discomfort.

Four milligrams of SDIO was dissolved in 150 *µ*L of injectable saline in a 1.5 mL Eppendorf vial and vortexed for 5 seconds to obtain a uniform solution. The solution was then passed through a 0.2-micron filter for sterilization and ready for injection. Animals were anesthetized under isoflurane, and SDIO was administered intravenously via tail vein with up to 150 *μ*L, 20 mg/kg of Fe, 24 h after intracerebral injection of TNF-*α*. Control animals were injected with the untargeted probe, DIO, of comparable composition to the experimental probe being tested. The injected dose was adjusted to match the same iron content as 20 mg/kg of animal.

### 2.6. MRI and Signal Processing

All images were acquired from a 7-Tesla (300 MHz) Bruker Avance Biospec system (Billerica, MA) equipped with a 95 mT/m max gradient set and 35 mm ID Doty coil, at ambient temperature (25°C). Anesthesia (isoflurane, inhalation, 1–3%) was administered during imaging acquisition. The animals were imaged 22 h after intracerebral injection of TNF-*α* and imaged again 4, 24, and 48 h after imaging probe injection (illustrated in Figure 1). A water heating pad was used on the animal holder and maintained at 37°C to prevent hypothermia. Each animal was imaged by T_2_^*∗*^-weighted sequences (FLASH 2D) with TR = 500 ms, TE = 5 ms, angle = 24°, NA = 8, FOV = 20 × 20 mm^2^, matrix = 128 × 128, slice thickness = 0.67 mm, and 12 slices.

Signal intensity and background noise for each 0.67 mm T_2_^*∗*^-weighted image slice was determined using Image J/Fiji software [31]. To compare particle uptake between time points and subjects, we calculated the contrast-to-noise ratio (CNR) generated by the probes on T_2_^*∗*^-weighted images. CNR was calculated as the difference in signal intensity between the injured site and the control region in the contralateral side of the brain scaled to image background noise. ROIs were drawn manually at the level of the injured site and were similar between all imaging time points.

### 2.7. Histology Analysis

Immediately following the final imaging time point at 48 h, the animals were anesthetized by high dose of anesthesia solution (Euthasol, 100 mg/kg, i.p.) followed by transcardial perfusion with phosphate-buffered saline (0.9%) and 10% buffered formalin phosphate solution. Brain tissue was dissected and postfixed in 10% buffered formalin phosphate solution at 4°C. Then, they were cryoprotected by incubation overnight in sucrose 30% solution. Forty-micrometer-thick coronal brain sections were cut on a freezing microtome (SM2400; Leica Microsystem, Wetzlar, Germany), and free-floating serial sections were preserved in a storing solution (glycerol 30%, ethylene glycol 30%, distilled water 30%, and PBS 10%) at −20°C until use.

Each brain was then processed for a double staining for microglia (anti-Iba-1 antibody) and iron (Perls' Prussian blue staining). Free-floating sections were rinsed in PBS 0.1 M and incubated with hydrogen peroxide 0.3% for 20 minutes. Pretreatment in a blocking solution for 30 minutes (PBS-Triton X-100, 0.2% (Sigma-Aldrich, MO, USA) and 4.5% normal goat serum) was performed before a 48-hour incubation, at +4°C, with the anti-Iba-1 antibody (Wako-019-19741, 1/1000 (Neuss, Germany)). Then, sections were incubated for 1 hour in secondary biotinylated anti-rabbit antibody (Vector Laboratories, 1/1000, Burlingame, CA, USA) before revelation. ABC Vectastain kit (Vector Laboratories, 1/250, Burlingame, CA, USA) was used for DAB revelation (DAB SK4100 kit, Vector Laboratories, Burlingame, CA, USA). The floating sections were then mounted on slides before Perls' staining, performed the next day. For Perls' staining, the slides were incubated in a methanol 20% and hydrogen peroxide 3% solution for 10 minutes. They were rinsed with distilled water and PBS 0.1 M before to be incubated with 2% potassium ferrocyanide (P9387, Sigma-Aldrich) and 2% hydrogen chloride solution for 20 minutes. The sections were finally dehydrated, covered by a cover slip, and digitized with a Zeiss AxioScan.Z1 (Oberkochen, Germany) whole-slide imaging microscope at a lateral resolution of 0.5 *μ*m.

### 2.8. Statistical Analysis

Unpaired two-tailed *t*-test was used to compare the means between different groups. A *t* statistic is calculated with *t* = difference in sample means/SE of difference in sample means. A *P* value is obtained by comparison with the t distribution on degrees of freedom. *P* < 0.05 was then considered significant. Nonparametric Mann–Whitney *U* test was also performed for the *in vivo* study (*N*=4) to compare with the parametric *t*-test.

## 3. Results

### 3.1. Syntheses and Characterization of DIO and SDIO

**Figure 2:**
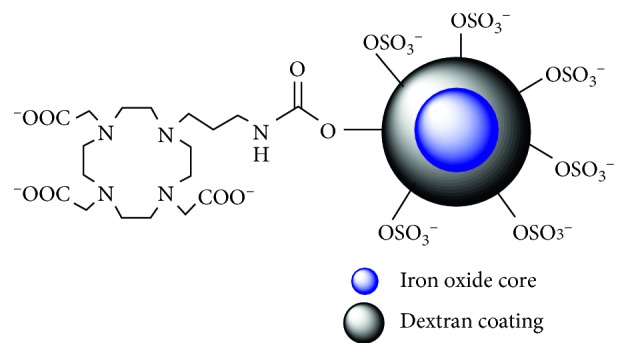
Schematic representation of SDIO.

The synthesis and physical characterization of both DIO and SDIO were reported in a previous article [30]. The properties of the materials synthesized for the current studies were confirmed to match to our published results. A schematic representation of SDIO is shown in Figure 2; DIO is the nonsulfated analog. Both types of nanoparticles had an iron oxide core size of 6 nm. The average hydrodynamic diameter of DIO and SDIO was around 20 and 60 nm, respectively. The increased size of SDIO was likely due to the repulsion between the negatively charged sulfate groups on the surface, which made the dextran coat expand. The iron content by total mass was 20% in DIO and 10% in SDIO. The zeta potential of the sulfated nanoparticles was −45 mV, while DIO had a positive charge of +11.7 mV in deionized water. Both nanoprobes had high relaxivities (DIO: *r*_1_ = 17.9 mM^−1^·s^−1^, *r*_2_ = 103.3 mM^−1^·s^−1^; SDIO: *r*_1_ = 14.2 mM^−1^·s^−1^, *r*_2_ = 72.8 mM^−1^·s^−1^), and the high *r*_2_/*r*_1_ ratios are indicative of good negative contrast agents. The decrease in relaxivity after sulfation could be due to the thicker coating of SDIO nanoparticles, which increases distance of the core from surrounding water, could reduce water exchange through the polysaccharide layer, and modulate relaxivity.

### 3.2. SDIO Is Biocompatible to Microglia

**Figure 3:**
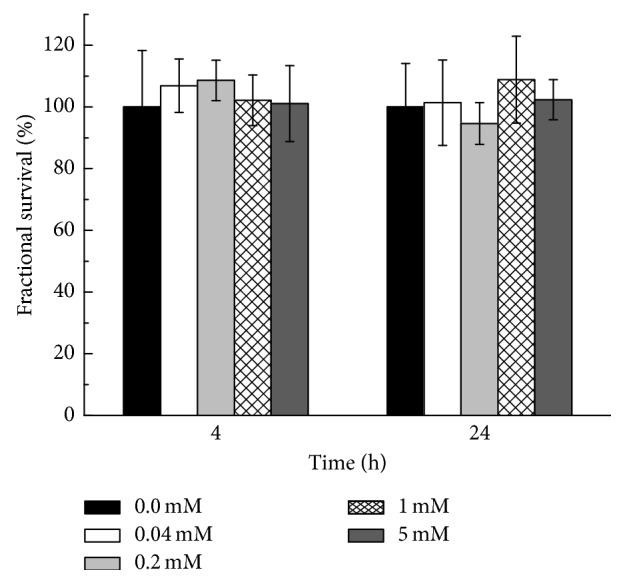
Cell viability studies with C_12_-resazurin assay. BV2 cells were incubated for 4 and 24 h with different iron concentrations of SDIO. Fluorescent intensities reflecting fractional survival were normalized against the signal from the untreated cells. All error bars present standard error of the mean (SEM) (*n*=3).

In our previous work, SDIO nanoparticles were evaluated on cultured liver cells (HepG2) and macrophages (J774 and P388D1) without observable toxicity. C_12_-resazurin viability assays were also used here to quantify cell viability on microglia. Cellular survival following incubation with increasing concentrations of SDIO is shown in Figure 3. Untreated cells (blank) served as the control. Fluorescent intensities, which reflected fractional survival of treated cells, were normalized against the signal intensities from the untreated cells. The average viability exceeds 95% after 4 or 24 h incubation with nanoparticles. An unpaired, two-tailed *t*-test was performed to compare treated cells against the control within each time point. There was no significant difference (*P* > 0.5) between the untreated cells and those incubated with SDIO at concentrations up to 5 mM, a concentration range relevant for *in vivo* MRI imaging.

### 3.3. High Cellular Uptake of SDIO by Microglia

**Figure 4:**
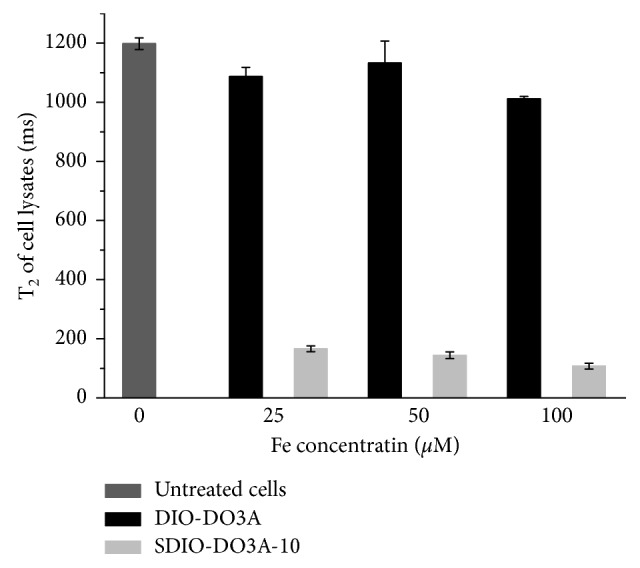
Specific uptake of SDIO nanoparticles by BV2 cells compared to nonsulfated analog DIO. Accumulation of iron oxide in cells reduced the transverse relaxation times of cell lysates. All error bars present SEM (*n*=3).

In our previous study with murine macrophages, sulfation of DIO facilitated SR-A targeting and enhanced microglial uptake. There was also a trend that uptake efficiency increased with higher sulfation levels on the nanoparticles. In this work, we identified the same trends when assessing the uptake of SDIO and DIO by microglia (BV2 cells) at 37°C. The T_2_ values of the cell lysates of microglia incubated with SDIO or DIO for 2 h are shown in Figure 4. There was significantly less uptake of the untargeted DIO (black bar) at 25, 50, and 100 *µ*M. With sulfation on the nanoparticles, the T_2_ decreased greatly for cells incubated with SDIO. *T*-test statistical calculations confirmed significant differences between the untreated cells and those incubated with SDIO (*P* < 0.001), while the difference between untreated cells and cells treated with DIO was not significant (*P* > 0.3), indicating that there was very little nonspecific uptake of nanoparticles by microglia. As the transverse relaxivity of SDIO (72.8 mM^−1^·s^−1^) was smaller than that of DIO (103.3 mM^−1^·s^−1^) due to the surface modification, there is actually an additional 1.4-fold increase in particle uptake efficiency for SDIO compared to that of DIO. This indicates that the surface sulfation significantly improved targeting to microglia and far exceeded the compensation for the decrease in relaxivities. This expected result suggested that more SDIO nanoparticles could accumulate in activated microglia when intravenously injected compared to injection of the same amount of the untargeted DIO.

### 3.4. *In Vivo* Visualization of Inflammation and Histological Confirmation

A mouse model of brain inflammation generated by unilateral intracerebral injection of TNF-*α* was previously reported in the literature [28]. TNF-*α* is a key regulator of the inflammatory response with its strong effect on vascular endothelium and endothelial leukocyte interactions [32]. In response to TNF-*α*, endothelial cells promote inflammation by displaying adhesion molecules for leukocyte recruitment. Here, to verify that intravenous administration of SDIO specifically detects neuroinflammation, we imaged the following groups: (1) TNF-*α*-only group (*n*=4), (2) IV-SDIO-only group (*n*=4), (3) TNF-*α* + IV-SDIO group (*n*=4), and (4) TNF-*α* + IV-DIO group (*n*=4).

**Figure 5:**
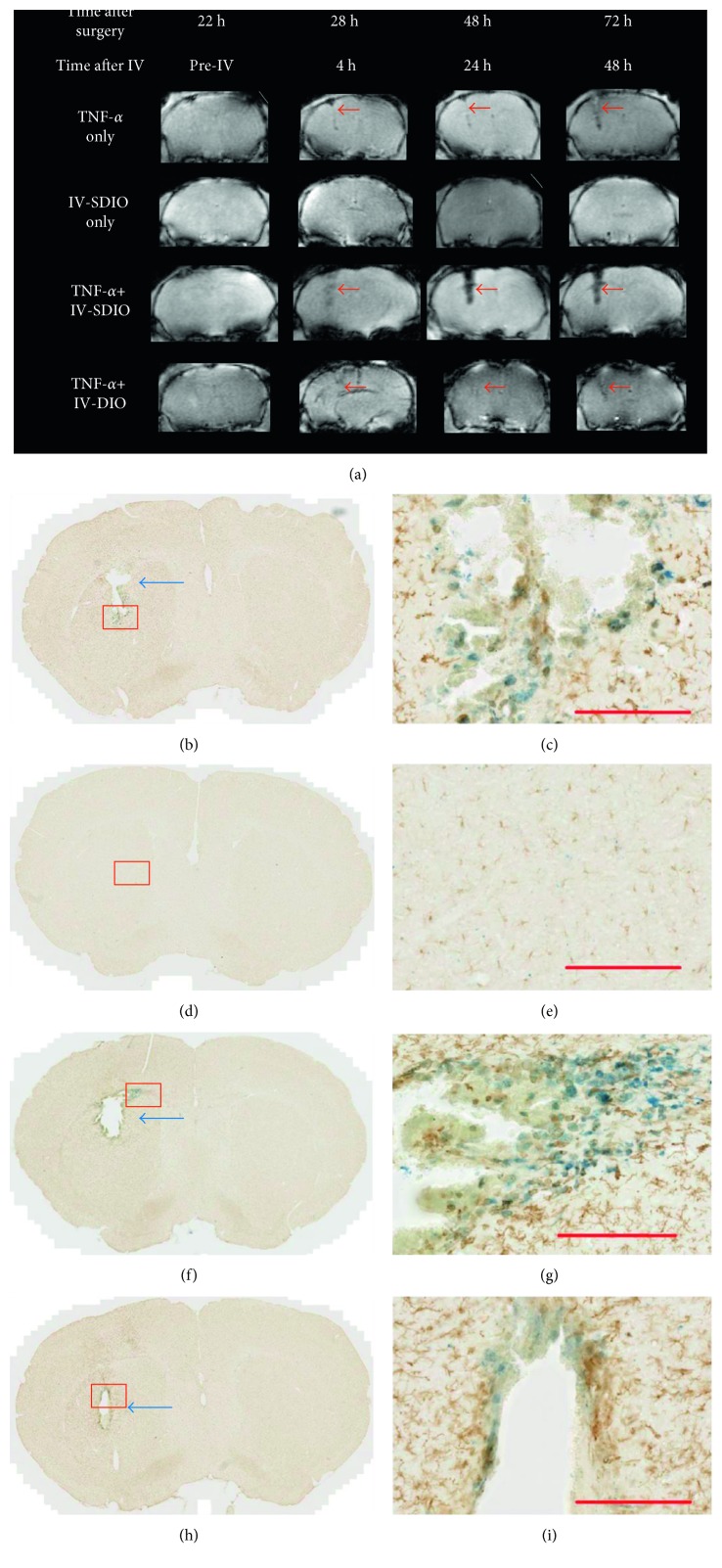
Representative T_2_^*∗*^-weighted MRI and histological staining of mouse brains from different imaging groups. (a) T_2_^*∗*^-weighted MR images in each row represent MRI scans at different time points from the same animal in that group. The second row is an animal that did not receive intracerebral injection, only intravenous SDIO injection. The other three rows are animals that underwent TNF-*α* intracerebral injections. From left to right, column 1 represents images after TNF-*α* injection, but before IV injection of contrast agent, the rest of the columns of MRI represent time points after intravenous injection of stated contrast agent (except for row 1, these animals were not injected with contrast agents). Red arrows denote the needle tracks and regions highlighted by contrast agents. (b–i) Histological evaluation of the same animals as shown in (a): (b, c) TNF-*α* only; (d, e) IV-SDIO only; (f, g) TNF-*α* + IV-SDIO; (h, i) TNF-*α* + IV-DIO. Blue arrows in (b), (f), and (h) denote small cavities in the brain that were observed. Images (c), (e), (g), and (i) show magnification of Iba-1 and iron staining of selected areas (red square) from images (b), (d), (f), and (h). Activated microglia at inflammation sites were stained brown by Iba-1 antibody, while iron oxide nanoparticles accumulated in the vicinity region were stained blue by Perls' Prussian blue. Scale bars represent 200 *µ*m.

As shown in Figure 5(a) (row 1), MR images of animals from the TNF-*α*-only group did not show much signal change 22 hours after TNF-*α* inoculation (before IV). The needle track at this time point was barely observable. MRI recorded 28 hours after surgery (4 h after IV) revealed only slightly enhanced negative contrast along the needle track (red arrows in Figure 5(a), row 1). These changes became more visible 24 and 48 hours after IV. This is probably due to increased inflammation and microhemorrhages with time. On the histological image, a small cavitation representing tissue loss was also detected. Anti-Iba-1 antibody staining confirmed the presence of activated microglia along the site of TNF-*α* inoculation (Figures 5(b) and 5(c)). A slight Perls' positive staining was also detected in the vicinity of the lesion, likely due to microhemorrhages along the needle track (Figures 5(b) and 5(c)).

MR images of the IV-SDIO-only control group (Figure 5(a), row 2) exhibited no accumulation of contrast in the intact brain as expected, suggesting that in the absence of cerebral inflammation and blood-brain barrier (BBB) disruption, the nanoparticles do not cross the BBB to accumulate in the brain. The histological staining (Figures 5(d) and 5(e)) did not demonstrate notable iron staining.

**Figure 6:**
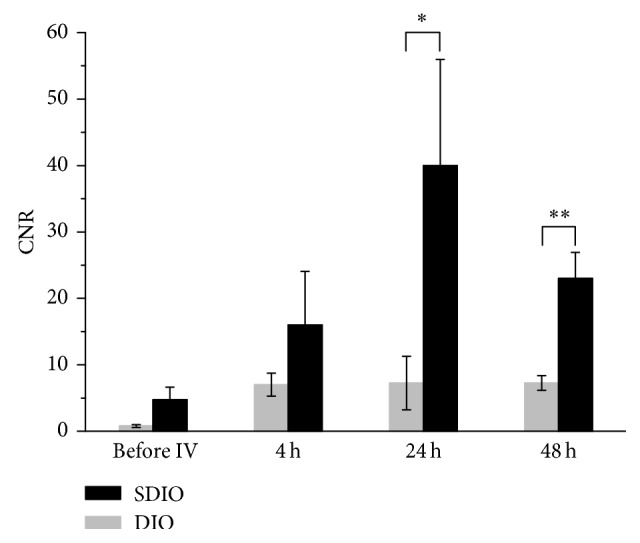
Average CNR of SDIO and DIO from MR images at different time points. All four animals from the experimental groups were calculated. ^*∗*^*P* < 0.05, ^*∗∗*^*P* < 0.01. All error bars present SEM (*n*=4).

We compared T_2_^*∗*^-weighted contrast of the glial scar produced by intracerebral TNF-*α* treatment groups and in groups that then received intravenous administration of either SDIO or DIO (Figure 5(a), rows 3 and 4, resp.). Twenty-two hours after unilateral (left) TNF-*α* inoculation, prior to contrast injection, no obvious signal changes could be detected at the site of the inoculation in any group (Figure 5(a), before IV). Four hours after IV injection of SDIO, the MR images displayed an area with strong negative contrast in the left side of the brain where TNF-*α* was injected (Figure 5(a), row 3). The region of T_2_^*∗*^ signal hypointensity was larger and through a greater brain depth in the SDIO group (row 3) than the TNF-*α* group that received DIO (row 4). There was no signal alteration in the control contralateral cortical hemisphere consistent with preferential uptake of the SDIO particles at the sites of focal inflammation. Quantitative analysis showed that CNR (calculated as the difference in signal intensity between the injured site and the control region in the contralateral side of the brain scaled to image background noise) reached 17 at 4 h post-IV injection scan (Figure 6). The absence of detectable signal changes in the MRI scan immediately after surgery and before SDIO injection (Figure 5(a)) indicates that the subsequent hypointensities following SDIO in the treated left hemisphere were a consequence of uptake of the iron oxide nanoparticles. Pathology did not demonstrate evidence of acute hemorrhage. The negative contrast was strongest at 24 h post-IV injection (Figure 5(a), row 3) and the CNR reached 49 (14-fold increase from the pre-IV scan) at this time point (Figure 6), which suggests higher accumulation of SDIO at the inflammatory site. The negative contrast then weakened slightly by 48 h post-IV injection and the CNR decreased to 26 (Figures 5(a) and 6).

For the TNF-*α* + IV-SDIO group, histological evaluations (Figures 5(f) and 5(g)) showed a small cavitation (blue arrow), a consequence of local cellular death following direct TNF-*α* instillation, representing a loss of dead or dying brain tissue during histological processing. TNF-*α* induced cavities were nonhemorrhagic and narrower in caliber than the corresponding broad T_2_^*∗*^ hypodensities visualized 24 and 48 h. Anti-Iba-1 antibody staining confirmed the presence of activated microglia at the site of TNF-*α* inoculation. We also found blue staining throughout the inflammatory lesion, indicating iron oxide nanoparticles accumulated in the injured hemisphere. The colocalization of the immunostaining and iron staining showed that iron oxide accumulated in microglia at the inflammatory sites and there was a strong correlation with the extent of iron staining to the contrast enhancement on the T_2_^*∗*^-weighted images.

Unlike the TNF-*α* + IV-SDIO group, the TNF-*α* + IV-DIO group did not present pronounced signal change after TNF-*α* inoculation (Figure 5(a) row 4 and Figure 6). Similar to the TNF-*α*-only group, the TNF-*α* + IV-DIO group displayed slightly enhanced negative contrast along the needle track but with very weak contrast compared to the TNF-*α* + IV-SDIO group, and the CNR in the TNF-*α* + IV-DIO group was under 10 for all time points. A significant difference between the TNF-*α* + IV-SDIO and TNF-*α* + IV-DIO conditions was reached 24 and 48 hours after the IV injection of the contrast agents (Figure 6, unpaired *t*-test *P* < 0.05). As the group size was small (*N*=4), we also performed Mann–Whitney *U* test to compare the CRN of IV-SDIO and IV-DIO at different time points. Mann–Whitney *U* is a nonparametric test that does not require the assumption of normal distribution and suits better for small sample size. *U* at 4 h, 24 h, and 48 h after IV was 4, 1, and 0, respectively. Similar to the *t*-test, a significant difference (*P* < 0.05) was also found between the TNF-*α* + IV-SDIO and the TNF-*α* + IV-DIO groups at 48 h post-IV injection. These results suggested inefficient uptake of DIO by microglia in the inflamed region. Histological results from the TNF-*α* + IV-DIO group also showed a small cavitation and activated microglia at the site of TNF-*α* inoculation (Figures 5(f) and 5(g)).

These results indicate that, with the same injected iron dose, SDIO accumulated preferentially compared to DIO particles at the sites of TNF-*α* inoculation, that is, neuroinflammation. Thus, sulfated nanoparticles are excellent, targeted contrast agents that enhance signal from microglia *in vivo* compared to conventional dextran-coated iron oxide. The result parallels that from the *in vitro* uptake studies (Section 3.3) where SDIO nanoparticles were more efficiently and avidly taken up by BV2 cells. The marked specificity of SDIO uptake by microglia is in contrast to SDIO uptake by macrophages that we have previously reported: microglia seem to show negligible uptake of the nontargeted DIO particles while macrophages showed reduced but measurable *in vivo* uptake of DIO [27]. The labeling of activated microglia by SDIO, thus, is comparatively much more selective than that of activated macrophages.

## 4. Discussion

MRI has shown great clinical utility for its high-resolution, noninvasive imaging of the brain without using ionizing radiation. Visualizing information about inflammatory processes such as activation of microglia can contribute to the understanding of the progression of neurotoxicity and cognitive impairment, facilitating development of improved treatments for neurodegenerative diseases. Previous studies have attempted to use endogenous iron deposits in activated microglia to generate contrast on MR images [33]. However, the poor signal from endogenous iron-labeled microglia limits the approach for a general assessment in the brain. Thus, various imaging agents have been under development to detect focal accumulations of activated microglia by MRI.

Fleig and colleagues previously reported labeling activated microglia with untargeted, ultrasmall superparamagnetic iron oxide (USPIO) nanoparticles in culture and in a rat glioma model [34]. Confocal microscopy of isolated brain sections showed localization of USPIO in microglia and macrophages. Although there is inflammatory activity that can sequester nontargeted agents, microglial uptake of USPIO was not efficient and transport was presumably through the enhanced permeability and retention effect. Previous MRI studies in animal models have used dextran-coated iron oxide doses up to 30 mg/kg body mass, 60 times the normal human dose (0.56 mg of iron/kg body weight [35]). This suggested that improved targeting and sequestration by microglia could reduce dose requirements.

Through SR-A, microglia interact with many components of cerebral inflammatory injury, such as oxidized low-density lipoprotein, amyloid fibrils, and apoptotic neurons [36, 37]. SR-A receptors have broad ligand specificity for a diverse array of polyanionic macromolecules, such as maleylated bovine serum albumin (mal-BSA), oxidized LDL, malondialdehyde-modified LDL, and polyinosinic acid [38–41]. The SR-A-targeted probes that we have developed indicate a significant improvement in the targeting and uptake of sulfated dextran-coated iron nanoparticles.

Here, we provide a first proof that SDIO labels *in vivo* microglia activated by intracerebral administration of TNF-*α*. In normal brain where the BBB is intact, entry of nanomaterials is very restricted, as passive diffusion of substances across the BBB endothelial cells is normally limited to small, lipophilic molecules less than 500 Da [42]. In this study, we wanted to focus on the targeting of microglia by SDIO and designed a TNF-*α*-based protocol inducing microglial activation associated with BBB disruption. SDIO but not the untargeted DIO particles showed significant labeling ability. This supports that labeling by SDIO is not simply due to microglial sequestration of material that passively diffused across the open BBB. First applications of SDIO without further modification of the contrast agents could thus target pathologies such as traumatic injuries, multiple sclerosis, or brain tumors in which the BBB is temporarily opened or chronically damaged [43].

Although it has long been known that BBB compromise commonly occurs in inflammation, infection, and all neurodegenerative disorders [44, 45], the damage to the BBB can be much milder than the trauma generated by intracerebral injections. Thus, SDIO may require modifications to penetrate the brain in applications where the BBB is not, or only slightly, opened. All nanoparticle-based agents face the challenge of penetrating the blood-brain barrier, and a number of methods described in the literature to overcome the BBB, such as the opening of tight junctions using osmotic pressure or ultrasound disruption, can effectively generate pores in the BBB that enable the entry of imaging agents or therapeutics to the brain [46, 47]. Future studies will need to modify SDIO and evaluate the improved formulation in models with milder BBB disruptions.

Transport of materials across the BBB is an active area of study by others, and previous works have demonstrated that coating nanoparticles with ligands (such as insulin or transferrin) or surfactants (such as polysorbate 80) could improve the passage through blood-brain barrier after systemic administration [48–50]. Surfactants can either directly disrupt the tight junctions or allow NPs absorbing to plasma protein, such as apolipoprotein E, to cross the BBB through endothelial cells by receptor-mediated transcytosis or endocytosis [51]. It has also been shown that magnetic heating (hyperthermia) of magnetic nanoparticles by a low radiofrequency field can increase BBB permeability without perturbing other brain cells [52]. This transient disruption of BBB is local and entirely reversible and could be potentially achieved by our SDIO nanoparticles. Methods that can keep SDIO in circulation longer and mediate transport across the BBB will greatly increase efficiency for detection of microglia in the brain and are topics for our future studies.

Future studies may also focus on the use of SDIO for other imaging methods. It is interesting to note that SDIO is conjugated with a macrocyclic chelator, which captures radionuclides including ^64^Cu, so that it could potentially be employed as a PET or PET/MRI multimodal agent [53]. PET is a very sensitive method, and SDIO-based contrast agents could be beneficial where low numbers of activated microglia are to be visualized or in diseases in which the crossing of BBB by SDIO is low.

## 5. Conclusions

We demonstrated the ability of SDIO targeting SR-A to image microglia actively involved in brain inflammation. The functionalized iron oxide nanoparticles were proven to be nontoxic and were efficiently internalized by activated microglia *in vitro* and *in vivo*. In a mouse model of brain inflammation, SDIO rendered much better *in vivo* contrast enhancement compared to the nonspecific, nonsulfated analog. These results support that SDIO is a promising MRI contrast agent, with potential multimodal function, to image activated microglia in inflammation and assess disease progression.

## Data Availability

Data of the study are available upon request.

## References

[1] Streit W. J., Mrak R. E., Griffin W. S. T. (2004). Microglia and neuroinflammation: a pathological perspective. *Journal of Neuroinflammation*.

[2] Chew L. J., Takanohashi A., Bell M. (2006). Microglia and inflammation: impact on developmental brain injuries. *Mental Retardation and Developmental Disabilities Research Reviews*.

[3] Giulian D. (1999). Microglia and the immune pathology of Alzheimer disease. *American Journal of Human Genetics*.

[4] Smith M. E. (2001). Phagocytic properties of microglia in vitro: implications for a role in multiple sclerosis and EAE. *Microscopy Research and Technique*.

[5] Town T., Tan J., Flavell R. A., Mullan M. (2005). T-cells in Alzheimer’s disease. *NeuroMolecular Medicine*.

[6] Weiner H. L., Frenkel D. (2006). Immunology and immunotherapy of Alzheimer’s disease. *Nature Reviews Immunology*.

[7] Perry V. H., Holmes C. (2014). Microglial priming in neurodegenerative disease. *Nature Reviews Neurology*.

[8] González H., Elgueta D., Montoya A., Pacheco R. (2014). Neuroimmune regulation of microglial activity involved in neuroinflammation and neurodegenerative diseases. *Journal of Neuroimmunology*.

[9] Wiley C. A., Lopresti B. J., Venneti S. (2009). Carbon 11–labeled Pittsburgh compound b and carbon 11–labeled (R)-PK11195 positron emission tomographic imaging in Alzheimer Disease. *Archives of Neurology*.

[10] Dickens A. M., Vainio S., Marjamäki P. (2014). Detection of microglial activation in an acute model of neuroinflammation using PET and radiotracers 11C-(R)-PK11195 and 18F-GE-180. *Journal of Nuclear Medicine*.

[11] Kreisl W. C., Fujita M., Fujimura Y. (2010). Comparison of [11C]-(R)-PK 11195 and [11C] PBR28, two radioligands for translocator protein (18 kDa) in human and monkey: implications for positron emission tomographic imaging of this inflammation biomarker. *NeuroImage*.

[12] Bloomfield P. S., Selvaraj S., Veronese M. (2015). Microglial activity in people at ultra high risk of psychosis and in schizophrenia: an [11C] PBR28 PET brain imaging study. *American Journal of Psychiatry*.

[13] Sandiego C. M., Gallezot J.-D., Pittman B. (2015). Imaging robust microglial activation after lipopolysaccharide administration in humans with PET. *Proceedings of the National Academy of Sciences*.

[14] Savitz S. I., Cox C. S. (2016). Concise review: cell therapies for stroke and traumatic brain injury: targeting microglia. *Stem Cells*.

[15] Owen D. R., Yeo A. J., Gunn R. N. (2012). An 18-kDa translocator protein (TSPO) polymorphism explains differences in binding affinity of the PET radioligand PBR28. *Journal of Cerebral Blood Flow and Metabolism*.

[16] Tronel C., Largeau B., Santiago Ribeiro M. J., Guilloteau D., Dupont A.-C., Arlicot N. (2017). Molecular targets for PET imaging of activated microglia: the current situation and future expectations. *International Journal of Molecular Sciences*.

[17] Toyama H., Hatano K., Suzuki H. (2008). In vivo imaging of microglial activation using a peripheral benzodiazepine receptor ligand: [11C] PK-11195 and animal PET following ethanol injury in rat striatum. *Annals of Nuclear Medicine*.

[18] Husemann J., Loike J. D., Anankov R., Febbraio M., Silverstein S. C. (2002). Scavenger receptors in neurobiology and neuropathology: their role on microglia and other cells of the nervous system. *Glia*.

[19] Limmon G. V., Arredouani M., McCann K. L., Minor R. A. C., Kobzik L., Imani F. (2008). Scavenger receptor class-A is a novel cell surface receptor for double-stranded RNA. *FASEB Journal*.

[20] Santiago-García J., Kodama T., Pitas R. E. (2003). The class A scavenger receptor binds to proteoglycans and mediates adhesion of macrophages to the extracellular matrix. *Journal of Biological Chemistry*.

[21] Abraham R., Singh N., Mukhopadhyay A., Basu S. K., Bal V., Rath S. (1995). Modulation of immunogenicity and antigenicity of proteins by maleylation to target scavenger receptors on macrophages. *Journal of Immunology*.

[22] Fong L. G., Le D. (1999). The processing of ligands by the class A scavenger receptor is dependent on signal information located in the cytoplasmic domain. *Journal of Biological Chemistry*.

[23] Józefowski S. (2012). The role of the class A scavenger receptors, SR-A and MARCO, in the immune system. Part 1. The structure of receptors, their ligand binding repertoires and ability to initiate intracellular signaling. *Postepy Higieny I Medycyny Doswiadczalnej*.

[24] Platt N., Haworth R., Darley L., Gordon S. (2002). The many roles of the class A macrophage scavenger receptor. *International Review of Cytology*.

[25] Jarrett B. R., Frendo M., Vogan J., Louie A. Y. (2007). Size-controlled synthesis of dextran sulfate coated iron oxide nanoparticles for magnetic resonance imaging. *Nanotechnology*.

[26] Jarrett B. R., Gustafsson B. R., Kukis D. L., Louie A. Y. (2008). Synthesis of 64Cu-labeled magnetic nanoparticles for multimodal imaging. *Bioconjugate Chemistry*.

[27] Tu C., Ng T. S., Sohi H. K. (2011). Receptor-targeted iron oxide nanoparticles for molecular MR imaging of inflamed atherosclerotic plaques. *Biomaterials*.

[28] Montagne A., Gauberti M., Macrez R. (2012). Ultra-sensitive molecular MRI of cerebrovascular cell activation enables early detection of chronic central nervous system disorders. *NeuroImage*.

[29] Goldstein J. L., Ho Y. K., Basu S. K., Brown M. S. (1979). Binding site on macrophages that mediates uptake and degradation of acetylated low density lipoprotein, producing massive cholesterol deposition. *Proceedings of the National Academy of Sciences*.

[30] Tang T., Tu C., Chow S. Y., Leung K. H., Du S., Louie A. Y. (2015). Quantitative assessment of binding affinities for nanoparticles targeted to the vulnerable plaque. *Bioconjugate Chemistry*.

[31] Schneider C. A., Rasband W. S., Eliceiri K. W. (2012). NIH Image to ImageJ: 25 years of image analysis. *Nature Methods*.

[32] Bradley J. (2008). TNF-mediated inflammatory disease. *Journal of Pathology*.

[33] Zeineh M. M., Chen Y., Kitzler H. H., Hammond R., Vogel H., Rutt B. K. (2015). Activated iron-containing microglia in the human hippocampus identified by magnetic resonance imaging in Alzheimer disease. *Neurobiology of Aging*.

[34] Fleige G., Nolte C., Synowitz M., Seeberger F., Kettenmann H., Zimmer C. (2001). Magnetic labeling of activated microglia in experimental gliomas. *Neoplasia*.

[35] Runge V. M. (2000). Safety of approved MR contrast media for intravenous injection. *Journal of Magnetic Resonance Imaging*.

[36] Xu Y., Qian L., Zong G. (2012). Class A scavenger receptor promotes cerebral ischemic injury by pivoting microglia/macrophage polarization. *Neuroscience*.

[37] Hooper C., Fry V. A., Sevastou I. G., Pocock J. M. (2009). Scavenger receptor control of chromogranin A-induced microglial stress and neurotoxic cascades. *FEBS Letters*.

[38] Horiuchi S., Sakamoto Y., Sakai M. (2003). Scavenger receptors for oxidized and glycated proteins. *Amino Acids*.

[39] Krieger M. (1994). Structures and functions of multiligand lipoprotein receptors: macrophage scavenger receptors and LDL receptor-related protein (LRP). *Annual Review of Biochemistry*.

[40] Gough P. J., Gordon S. (2000). The role of scavenger receptors in the innate immune system. *Microbes and Infection*.

[41] Platt N., Gordon S. (2001). Is the class A macrophage scavenger receptor (SR-A) multifunctional?—The mouse’s tale. *Journal of Clinical Investigation*.

[42] Wohlfart S., Gelperina S., Kreuter J. (2012). Transport of drugs across the blood–brain barrier by nanoparticles. *Journal of Controlled Release*.

[43] Price L., Wilson C., Grant G. (2016). *Blood–Brain Barrier Pathophysiology following Traumatic Brain Injury*.

[44] Obermeier B., Daneman R., Ransohoff R. M. (2013). Development, maintenance and disruption of the blood-brain barrier. *Nature Medicine*.

[45] Erickson M. A., Dohi K., Banks W. A. (2012). Neuroinflammation: a common pathway in CNS diseases as mediated at the blood-brain barrier. *Neuroimmunomodulation*.

[46] Zhang T.-T., Li W., Meng G., Wang P., Liao W. (2016). Strategies for transporting nanoparticles across the blood–brain barrier. *Biomaterials Science*.

[47] Santin M. D., Debeir T., Bridal S. L., Rooney T., Dhenain M. (2013). Fast in vivo imaging of amyloid plaques using μ-MRI Gd-staining combined with ultrasound-induced blood–brain barrier opening. *NeuroImage*.

[48] Wiley D. T., Webster P., Gale A., Davis M. E. (2013). Transcytosis and brain uptake of transferrin-containing nanoparticles by tuning avidity to transferrin receptor. *Proceedings of the National Academy of Sciences*.

[49] Kreuter J., Petrov V., Kharkevich D., Alyautdin R. (1997). Influence of the type of surfactant on the analgesic effects induced by the peptide dalargin after its delivery across the blood–brain barrier using surfactant-coated nanoparticles. *Journal of Controlled Release*.

[50] Petri B., Bootz A., Khalansky A. (2007). Chemotherapy of brain tumour using doxorubicin bound to surfactant-coated poly (butyl cyanoacrylate) nanoparticles: revisiting the role of surfactants. *Journal of Controlled Release*.

[51] Saraiva C., Praça C., Ferreira R., Santos T., Ferreira L., Bernardino L. (2016). Nanoparticle-mediated brain drug delivery: overcoming blood–brain barrier to treat neurodegenerative diseases. *Journal of Controlled Release*.

[52] Tabatabaei S. N., Girouard H., Carret A.-S., Martel S. (2015). Remote control of the permeability of the blood–brain barrier by magnetic heating of nanoparticles: a proof of concept for brain drug delivery. *Journal of Controlled Release*.

[53] Jarrett B. R., Correa C., Ma K. L., Louie A. Y. (2010). In vivo mapping of vascular inflammation using multimodal imaging. *PLoS One*.

